# Interaction of stomatal behaviour and vulnerability to xylem cavitation determines the drought response of three temperate tree species

**DOI:** 10.1093/aobpla/plz058

**Published:** 2019-09-23

**Authors:** Zhicheng Chen, Shirong Liu, Haibo Lu, Xianchong Wan

**Affiliations:** 1 Institute of New Forestry Technology, Chinese Academy of Forestry, Beijing, China; 2 Key Laboratory of Forest Ecology and Environment of State Forestry Administration, Institute of Forest Ecology, Environment and Protection, Chinese Academy of Forestry, Beijing, China

**Keywords:** Anisohydric, carbon starvation, climate change, hydraulic failure, isohydric, xylem vulnerability

## Abstract

How the mortality and growth of tree species vary with the iso-anisohydric continuum and xylem vulnerability is still being debated. We conducted a precipitation reduction experiment to create a mild drought condition in a forest in the Baotianman Mountains, China, a sub-humid region. Three main sub-canopy tree species in this region were examined. After rainfall reduction, *Lindera obtusiloba* showed severe dieback, but two other co-occurring species did not show dieback. The water potential at stomatal closure of *Dendrobenthamia japonica*, *L. obtusiloba* and *Sorbus alnifolia* was −1.70, −2.54 and −3.41 MPa, respectively, whereas the water potential at 88 % loss in hydraulic conductivity of the three species was −2.31, −2.11 and −7.01 MPa, respectively. Taken together, near-anisohydric *L. obtusiloba* with vulnerable xylem was highly susceptible to drought dieback. Anisohydric *S. alnifolia* had the most negative minimum water potential, and its xylem was the most resistant to cavitation. Isohydric *D. japonica* conserved water by rapidly closing its stomata. Ultimately, the hydraulic safety margin (HSM) of *L. obtusiloba* was the smallest among the three species, especially in precipitation-reduced plots. In terms of the stomatal safety margin (SSM), *L. obtusiloba* was negative, while *S. alnifolia* and *D. japonica* were positive. Of the two species without dieback, rainfall reduction decreased growth of *D. japonica*, but did not influence growth of *S. Alnifolia*; meanwhile, rainfall reduction led to a decrease of non-structural carbohydrates (NSCs) in *D. japonica*, but an increase in *S. alnifolia*. It is concluded that HSM as well as SSM allow interpreting the sensitivity of the three sub-canopy species to drought. The drought-induced dieback of *L. obtusiloba* is determined by the interaction of stomatal behaviour and xylem vulnerability, and the species could be sensitive to climate change-caused drought although still in sub-humid areas. The isohydric/anisohydric degree is associated with NSCs status and growth of plants.

## Teaser

Climate change-induced drought has caused increasing plant mortality around the globe. In this study, we artificially reduced annual rainfall in the field of temperate continental mountains, and investigated different responses of three understory tree species to drought and their response strategies. This study aimed to explore how future climate change-caused droughts might affect vegetation succession in the region. Plants have two important attributes that regulate water balance: closable pores in leaves (stomata) and long-distance water transport channels (xylem conduits). It was found that the synergistic effect of stomatal adjustment and xylem anti-embolism ability was the key to drought resistance.

## Introduction

Climate change-induced drought events have caused increasing plant mortality around the globe (Allen *et al.* 2010; Hartmann *et al.* 2015; Anderegg *et al.* 2016), and future climate trends are predicted to lead to greater drought-related impacts on forest ecosystems (IPCC 2013). Improved understanding of cross-species mortality patterns under drought conditions is helpful to predict the impacts of climate change on biodiverse ecosystems (Anderegg *et al.* 2016). Thus, research on drought physiological responses of woody plants has recently become increasingly important. Hydraulic failure and carbon starvation are two main mechanisms for explaining drought-induced mortality of woody plants (McDowell *et al.* 2008). The hydraulic failure theory states that reduced soil water supply coupled with high evaporative demand can cause the plant vascular hydraulic transport system to lose hydraulic conductivity (meaning it becomes air-filled), leading to desiccation of the shoot or the whole tree (McDowell *et al.* 2008; Meinzer and McCulloh 2013; Choat *et al.* 2018). The carbon starvation hypothesis considered that stomatal closure to prevent hydraulic failure causes photosynthetic carbon uptake to diminish, with the plant starving as a result of continued carbohydrate demand by respiration, growth and defence (McDowell *et al.* 2008; McDowell 2011; Adams *et al.* 2017).

Across the continuum of stomatal regulation of water status, plants can be classified as either isohydric or anisohydric (Tardieu and Simonneau 1998; McDowell *et al.* 2008). The stomatal conductance versus leaf water potential curves over many species formed a continuum, rather than dichotomy distribution between isohydric and anisohydric (Klein 2014; Lavoie-Lamoureux *et al.* 2017). Isohydric plants reduce stomatal conductance (*g*_s_) rapidly as soil water potential decreases or as atmospheric conditions dry, thereby restricting excessive water loss and maintaining high plant water potential, albeit at the cost of carbon assimilation. Anisohydric species, in contrast, maintain open stomata and allow midday leaf water potential to decline with decreasing soil water potential to maintain CO_2_ uptake (McDowell *et al.* 2008; Woodruff *et al.* 2015). In other words, isohydry is dehydration avoidance, while anisohydry is dehydration tolerance (Lavoie-Lamoureux *et al.* 2017). Theoretically, isohydric species are more likely to succumb to carbon starvation during protracted mild drought, whereas anisohydric species are more likely to die from hydraulic failure during short but severe droughts (e.g. Piñon–juniper woodlands of southwestern USA) (McDowell *et al.* 2008; Dickman *et al.* 2015). This iso-anisohydric theory thus constructs a framework representing the response of plants to drought-induced hydraulic failure and carbon starvation. However, the theory cannot tell which mechanism has advantage in terms of plant survival to drought stress. For example, within the same habitat, the mortality of anisohydric species can be less during extreme droughts (McDowell *et al.* 2008; Dickman *et al.* 2015; Woodruff *et al.* 2015), while the species occupying middle positions along the isohydric to anisohydric continuum suffered less mortality than those at either extremes (Gu *et al.* 2015). Lately, through data review, Martínez-Vilalta and Garcia-Forner (2017) come to the conclusion that iso/anisohydry defined in terms of Ψ _L_ regulation cannot be used as an indicator of a specific mechanism of drought-induced mortality or as a proxy for overall plant vulnerability to drought. In addition, it is noteworthy that the isohydric or anisohydric is not an intrinsic property of the plant itself, but changes with the environment (Hochberg *et al.* 2018). Thus, it may be limited to species comparison in the same habitat.

The indefinite relationship between tree mortality and the iso-anisohydric continuum may result from xylem vulnerability. Many previous studies showed that xylem vulnerability plays an important role in determining drought tolerance (McDowell *et al.* 2008; Breshears *et al.* 2009; Choat *et al.* 2012; Skelton *et al.* 2015). The most common index of xylem vulnerability of embolism resistance for is the P_50_ and P_88_ (water potential at 50 and 88 % loss of conductivity). Hydraulic failure leading to irreversible drought-induced dysfunction in angiosperm trees usually occurs at a very high level of stem xylem embolism, being close to 88 % (Brodribb and Cochard 2009; Brodribb *et al.* 2010; Johnson *et al.* 2012; Urli *et al.* 2013). Thus, in this study, both P_50_ and P_88_ are the metric used to define the safety margin. The difference between the typical minimum water potential (Ψ _min_) and the embolization threshold is regarded as hydraulic safety margin. With the framework of hydraulic safety margin, Choat *et al.* (2012) found that 70 % of 226 forest species operate with narrow (<1 MPa) hydraulic safety margins and therefore predicted the species would face long-term reductions in productivity and survival with global climate change whether the species are distributed in arid or humid areas. A meta-analysis across 475 species performed by Anderegg *et al.* (2016) found that the hydraulic safety margin was the best predictor of the patterns of cross-species mortality.

At present, it has been suggested that isohydric species tend to have more vulnerable xylem but a stronger capability to repair xylem cavitation compared with more anisohydric species (Meinzer and McCulloh 2013; Brodribb and McAdam 2015; Dickman *et al.* 2015; Woodruff *et al.* 2015). However, whether different stomatal behaviour is associated with different xylem vulnerability is still unclear (Skelton *et al.* 2015). Therefore, more empirical data are needed to conclude whether isohydric species have a greater hydraulic safety margin or more vulnerable xylem in comparison to that of anisohydric species.

It is challenging to determine whether the reason for drought-induced plant mortality is hydraulic failure, carbon starvation or their interaction. The relationship among Ψ _min_, water potential at stomatal closure (P_g88_) and embolization threshold may provide a comprehensive framework. Skelton *et al.* (2015) defined the difference between P_g88_ and embolization threshold as the stomatal safety margin; large positive values indicate that stomatal closure occurred before large losses of conductivity, whereas negative values indicate that stomatal closure is subsequent to embolization threshold. Therefore, the stomatal safety margin represents the degree of stomatal regulation over cavitation.

There are two naturally occurring isotopes of carbon, ^12^C and ^13^C. The overall abundance of ^13^C relative to ^12^C in C_3_ plant tissues is commonly less than in the carbon of atmospheric CO_2_, that is, the incorporation of CO_2_ into plant biomass occurs a discrimination against ^13^C (Farquhar *et al.* 1989). However, under drought condition, stomatal closure results in an increase in the isotope carbon isotope composition (δ ^13^C) that is often used for studying water use of trees, because the stable isotope composition can directly reflect water-use efficiency (WUE) of trees (Farquhar *et al.* 1982). On the other hand, the stomatal conductance is related to WUE of trees, that is, the iso-anisohydric behaviour may also be related to WUE.

As mentioned above, isohydric species are prone to depletion of non-structural carbohydrates (NSCs) due to their frequent and easy closure of stomata under drought stress, which depresses photosynthesis and carbon accumulation. On the contrary, anisohydric species can to a certain degree maintain their stomata aperture and photosynthesis under drought stress. Thus, NSC could be an indicator to express isohydric/anisohydric degree. However, the NSC content has been shown to increase, decrease or remain unchanged during drought (Sala and Hoch 2009; Woodruff and Meinzer 2011; Anderegg and Anderegg 2013; Gaylord *et al.* 2013; Dickman *et al.* 2015; Woodruff *et al.* 2015). The NSC storage dynamics under drought conditions are probably related to the timing and magnitude of the coupling between water stress and the supply and demand of NSC. Because of the greater sensitivity to water stress of turgor-driven cell expansion than photosynthesis, moderate water stress generally leads to greater reductions in growth than photosynthesis and, ultimately, NSC concentrations increase (Woodruff and Meinzer 2011). However, if drought persists and/or becomes more severe, causing significant reductions in photosynthesis for a sufficiently long period, the previously accumulated NSCs can be depleted as a result of ongoing metabolism and other demands on NSC reserves (McDowell 2011). However, in the literature related to drought, NSCs and growth data are scarce.

In April 2013, we established precipitation-reduced plots by covering 50 % of the plot area with interception roof in a natural secondary oak (*Quercus* spp.) forest in the Baotianman Natural Reserve (111°47′–112°04′E, 33°20′–33°36′N) of China, simulating precipitation reductions caused by global climate change **[see****Supporting Information—Fig. S1****]**. Northern and north-central China experienced a decrease in mean annual precipitation between 1960 and 2010 (Wang *et al.* 2015) and growing season precipitation is expected to decrease further until 2035 in parts of central China (IPCC 2013). The experimental plots were originally designed to investigate the effects of experimental throughfall reduction on soil carbon dynamics in a warm-temperate oak forest, and other eco-physiological study. There are three main deciduous sub-canopy tree species in the forest. At the end of August 2014, we found that a deciduous sub-canopy tree species, *Lindera obtusiloba*, showed dieback in rainfall-intercepted plots **[see****Supporting Information—Fig. S2****]**, while those in control plots grew normally. The other two co-occurring deciduous sub-canopy tree species, *Dendrobenthamia japonica* and *Sorbus alnifolia*, did not exhibit dieback in both precipitation-reduced plots and control plots. The differential responses of the three species to the precipitation reduction have aroused our interest to study the mechanisms of drought-induced dieback of the woody plants in the region. In addition, we were interested in how the natural hydraulic traits endow the species to cope with the climatic change-caused drought. The main objectives of this study were to investigate: (i) the stomatal behaviour of the three species, and how it is associated with the xylem vulnerability, (ii) whether the rainfall reduction causes dieback of the tree species, and what the reason for the dieback is, (iii) how the stomatal regulation in response to water potential variation affects NSC and growth of the three species in the rainfall reduction condition.

## Materials and Methods

### Study site and materials

This study was conducted at the Forest Ecological Research Station in the Baotianman Natural Reserve (111°47′–112°04′E, 33°20′–33°36′N), Henan Province, China. The average elevation is 1400 m. The region is characterized as a typical temperate continental monsoon climate. The mean annual precipitation is ~900 mm, which occurs mainly in summer (June–September, 60 %). The mean annual air temperature was 15.1 °C, with the lowest and highest temperatures being −14.8 and 41.2 °C, respectively. The mean relative humidity was 68 %. Upland soils were dominated by mountain yellow brown soil (Chinese classification). The study site was dominated by the canopy species *Quercus aliena* var. *acuteserrata*. Our study species, *D. japonica*, *L. obtusiloba* and *S. alnifolia*, were associated species.

### Experimental design

Six 20 × 20 m plots were set in the Natural Reserve in April 2013, and three plots were subjected to natural precipitation and served as control. The other three plots were treated with partial rainfall exclusion by intercepting the rainfall **[see****Supporting Information—Fig. S1****]**. In the rainfall-intercepted plots, throughfall was reduced by ~50 % relative to that of the natural plots using 160 pieces of transparent plastic film (0.5 × 3 m) fixed to an ~1.5–2.5 m stainless steel frame per rainfall-intercepted plot; the intercepted rainfall was diverted by guiding gutters. Water-prevention sheets were buried around the intercepted rainfall plots by 1 m depth for preventing water exchange between the inside and outside of the plots. There were 27 *D. japonica*, 47 *L. obtusiloba* and 29 *S. alnifolia* trees in the rainfall-intercepted plots, and there were 28 *D. japonica*, 35 *L. obtusiloba* and 30 *S. alnifolia* trees in the control plots. In 2013, the DBH (diameter at breast height) values (mean ± SE) of *D. japonica*, *L. obtusiloba* and *S. alnifolia* were 3.26 ± 0.40, 3.93 ± 0.25 and 3.35 ± 0.48 cm, respectively, and they were all 15- to 20-year-old. There were no significant differences in DBH for every species between control and reduced-rainfall plots. The height of all trees of study species was 3–6 m. *Dendrobenthamia japonica* and *L. obtusiloba* are diffuse-porous, while *S. alnifolia* is ring-porous. We arbitrarily set a criterion for judgement of dieback: the leader or three and more branches’ tips are dead.

All the measurements in this study were conducted in 2014–16. Predawn and midday twig water potentials, gas exchange and NSCs of *D. japonica*, *L. obtusiloba* and *S. alnifolia* were measured four times each during June 2014, August 2014, July 2015 and September 2015 in rainfall-intercepted plots and control plots. Moreover, leaf stomatal conductance-water potential curves, xylem vulnerability curves (VCs) to cavitation and shoots pressure-volume (P-V) curves of the three species were measured in June 2016. The final growth and the xylem δ ^13^C measurement were done in 2016. Sample size was six replicates (plants or soil sites), which were randomly taken from three plots in each precipitation manipulation treatment. The precipitation manipulation treatments were considered a fixed effect, with study plots as random factors. The P_88_, P_g88_ and the water potential at the turgor loss point (Ψ _TLP_) were measured with the plants in control plots, because we were interested in how the natural hydraulic traits relate to the climatic change-caused drought. The other parameters were comparatively measured between throughfall exclusion and control.

### Soil moisture measurement

Dynamics of soil moisture (volumetric water content, V/V) at the 5–10 cm soil layer in control and rainfall-intercepted plots were determined using an Em50 Series Data Collection System (Decagon Devices, Inc., USA) from August 2013 to July 2016. Four soil moisture probes were installed at random locations in each plot. Data were automatically collected every 30 min.

### Predawn and midday water potential, and gas exchange

Predawn and midday twig water potentials were measured using a portable pressure chamber (Model No. 1000; PMS Instruments Co., Corvallis, OR, USA) on sunny days. The predawn water potential (Ψ _pd_) was measured around 0530 h, while the midday water potential (Ψ _md_) was measured between 1130 and 1330 h. Water potential and gas exchange were always determined within 3 days to increase the interspecific comparability.

Gas exchange was measured with an LI-6400XT portable photosynthesis system (Li-Cor Inc., Lincoln, NE, USA) on sunny days. The measurements were made between 0900 and 1130 h. Three sun-exposed leaves per tree were selected for measurements. Measurements were taken using a standard 2 cm × 3 cm chamber equipped with blue-red light emitting diodes providing a photosynthetic photon flux density of 1500 µmol m^−2^ s^−1^. The ambient CO_2_ concentration was maintained at 400 ppm. The chamber was maintained at air temperature of 26 °C and relative humidity of 60 %. Both water potential and gas exchange were measured on six individual trees at random each time for each species in control and rainfall-intercepted plots.

### Xylem VCs and hydraulic safety margin

Xylem VCs were constructed by plotting stem percentage loss of hydraulic conductivity (PLC) against their corresponding xylem tension (ψ) values via bench dehydration (Sperry *et al.* 1988; Cochard *et al.* 2013). To conveniently determine the VCs, branches were collected at predawn after a rainfall event. The maximum xylem vessel length for each species was measured before the VC determination in order to avoid the introduction of errors when cutting branches for PLC measurements (Wheeler *et al.* 2013). The maximum vessel length was measured using the ‘injection air method’ described by Cohen *et al.* (2003) and Wang *et al.* (2014). The maximum vessel lengths for *D. japonica*, *L. obtusiloba* and *S. alnifolia* were 52 ± 2.5, 60 ± 3.3 and 19.5 ± 1.2 cm, respectively. Thus, it was ensured that the branches collected for VCs measurement were longer than the maximum vessel length with forks. Branches were collected at predawn after a rainfall event and immediately enclosed in plastic bags for transport to the lab within a half hour. Branches were dehydrated on bench at ambient irradiance and temperature in order to obtain a series of xylem tensions over a range of PLC. Branches were carefully wrapped in aluminium foil once they reached the desired tension. After >1 h of equilibration, a short leafy shoot was cut in air from one fork of each branch at a distance of at least one maximum vessel length from the sample segment. The purpose of this procedure was to determine the xylem tension with the pressure chamber. Soon after excising the short shoot, two to four segments (10–15 cm long) of 1- or 2-year-old branches used for PLC determination were cut under water and then measured with a Sperry apparatus (Sperry *et al.* 1988). The apparatus consists of a PEEK tubing system connected with three-way stopcocks, solution supply reservoir, a compressed air tank with regulator, an electronic balance and a computer. The upstream is connected to the solution supply reservoir or the compressed air tank, and the downstream is connected to the balance which interfaces with a computer. The apparatus was cleaned prior to PLC measurements. The system was prefilled with ultra-pure, degassed 25 mM KCl solution passed through a 0.2-μm filter. The initial hydraulic conductivity (*K*_i_) was measured gravimetrically by determining the flow rate of the KCl solution at a pressure differential of 4 kPa. The stem segment was then flushed for 10 min in order to remove air embolisms at a pressure of 0.08, 0.15 and 0.175 MPa for *D. japonica*, *L. obtusiloba* and *S. alnifolia*, respectively. The pressures and flushing duration are determined based on a preliminary experiment. The hydraulic conductivity was then determined again at a pressure differential of 4 kPa and set as the maximum hydraulic conductivity (*K*_max_). The PLC was calculated as follows:

$$\begin{array}{l} PLC=100\times (K_{\max}-K_{i})/K_{\max} \end{array}$$

To acquire enough data to construct the complete VC, >30 branches from >8 trees of each species were measured. The VCs were fitted by following the equation below (Pammenter and Vander Willigen 1998):

$$\begin{array}{l} PLC=100/(1+\exp(a(\psi -b))) \end{array}$$

where ψ is the water potential. Then, the P_50_ and P_88_ values (the water potential at which 50 and 88 % of hydraulic conductivity were lost, respectively) of the three species were obtained. The hydraulic safety margin of each species was the difference between the measured minimum water potential and P_88_ and P_50_ as well.

### Leaf stomatal conductance-water potential curves and stomatal safety margin

Leaf stomatal conductance declines when water potential decreases. This curve was determined after a rainfall event because the water potential of the branches needs to be high, similar to the requirements for VC determination. In addition, stomatal conductance is also influenced by illumination; therefore, this curve was determined after 0900 h, at which point photosynthetic induction had been occurring for 2 h on sunny days, ensuring leaf stomata had fully opened. The leaf stomatal conductance and leaf water potential were measured on cut branches under sunlight. The leaf stomatal conductance was first measured using LI-6400XT, after which the leaf was removed and the leaf water potential was measured using the PMS1000 pressure chamber. Leaf stomatal conductance-water potential curves were then constructed by plotting stomatal conductance against the corresponding leaf water potential and were fitted by following the equation below (Brodribb and Cochard 2009):

$$\begin{array}{l} g_{s}=a/(1+\exp(-(\psi -x_{0})/b)) \end{array}$$

where ψ represents the leaf water potential, *g*_s_ represents the stomatal conductance, and *a*, *x*_0_ and *b* are constants. The water potentials at 50 % stomatal closure of the three species were obtained by the curves. The water potentials at a proxy of the point of stomatal closure (P_g88_) of the three species were determined at 88 % stomatal closure or 12 % of maximum stomatal conductance. The maximum *g*_s_, used for calculating percentage stomatal closure, was found by extrapolating to where the curve reaches ψ = 0. The stomatal safety margin was the margin between P_g88_ and P_88_ as well as P_50_. More than 10 branches from >6 trees were measured for the curve of each species.

### Water potential at the turgor loss point

Twig water potentials at the turgor loss point (Ψ _TLP_) of the three species were determined by P-V curves (Tyree and Hammel 1972). The branches for the P-V curve measurements were also sampled at predawn after a rainfall event. The sampled branches were inserted into water and covered with black plastic bags to make the water potential more than −0.03 MPa. The twigs or leaves were cut off to measure the fresh weight, and then determine the water potential immediately to obtain the corresponding water potential and weight by natural dehydration. After the measurements, the materials were dried in the oven to obtain dry weight which was used to calculate the relative water content of the series. Finally, the P-V curve was obtained. Sample weights versus balance pressures were measured >10 times **[see****Supporting Information—Fig. S3****]**. Sample pressures were measured by pressure chamber (Model No. 1000; PMS Instruments Co., Corvallis, OR, USA). Ψ _TLP_ values were used as the means of P-V curves, which were determined on four to six individual plants. The linear part of the P-V curve was used to fit a linear equation. The 1/RWC (relative water content) values of the curvilinear part were substituted into the equation to obtain a series of corresponding osmotic potentials. Water potentials minus the corresponding osmotic potential were the turgor pressure values. The turgor pressures were regressed with the 1/RWC values of the straight part of the P-V curve to obtain the 1/RWC value at the zero turgor, and the 1/RWC value was substituted into the previous equation to obtain the water potential at the turgor loss point (Ψ _TLP_).

### Chemical analyses

In order to avoid the daily fluctuations of NSC concentrations in plant tissues, samples for NSC measurements were collected at the same time as the determination of predawn water potential. The leaf and xylem samples used for soluble sugar (SS) and starch (St) measurements were heated at 105 °C for 20 min to stop all enzymatic activity and then oven-dried at 75 °C for 48 h. All dried samples were ground and sieved through a 100-mesh screen and the powder was used for the determination of SS and St content by the anthrone-sulfuric acid method (Hansen and Moller 1975). The SSs were first extracted three times from the ground samples using 80 % ethanol at 80 °C. The residue obtained after extraction was analysed for St content by digestion using perchloric acid solution. Following extraction, the concentrations of SSs and St were determined photometrically in the presence of anthrone-sulfuric acid reagent on a 96-well microplate photometer (Model SpectraMax 190; Molecular Devices Co., San Francisco, CA, USA). The sum of SSs and St is referred to as the total NSCs. Samples of every time determination of NSCs were from six randomly selected individuals per species both in the control and rainfall-intercepted plots.

The precipitation manipulation experiment in this study started in April 2013. To investigate water use over that period of time, 3-year-old branches were sampled for xylem δ ^13^C measurement in September 2016. The xylem δ ^13^C samples were collected from six individuals and were measured using an isotope ratio mass spectrometer (DELTA V Advantage, Thermo Fisher Scientific, Inc., USA), and the analytical precision of δ ^13^C was ±0.1‰.

### Growth

Shoot extensions of the three species in the control and rainfall-intercepted plots were monitored from May 2015 to September 2016 using a vernier calliper on 12 shoots from six individuals. The DBH increments from April 2013 to September 2016 were measured with a tree diameter ruler on each individual in the plots.

### Statistical analyses

Independent-sample *t*-tests were conducted to detect differences in water potential, gas exchanges and NSCs, as well as shoot extension and DBH increments of each species between control plots and rainfall-reduced plots. The differences of xylem δ ^13^C were analysed using two-way ANOVA (with species and treatments as factors). All analyses were performed using SAS v10 software (SAS Institute Inc., Cary, NC, USA). Comparisons were considered significant at the 95 % confidence level. Correlations between leaf stomatal conductance and water potential; and P_g88_, P_88_ and Ψ _TLP_ were fitted using SigmaPlot (version 12.5, Systat Software, San Jose, CA, USA). Sample size was six replicates (plants or soil sites), which were randomly taken from three plots in each treatment. The treatments of the rainfall reduction and the natural control were considered a fixed effect, with study plots as random factors. Figures were constructed with SigmaPlot.

## Results

### Soil moisture and tree dieback

**Figure 1.**
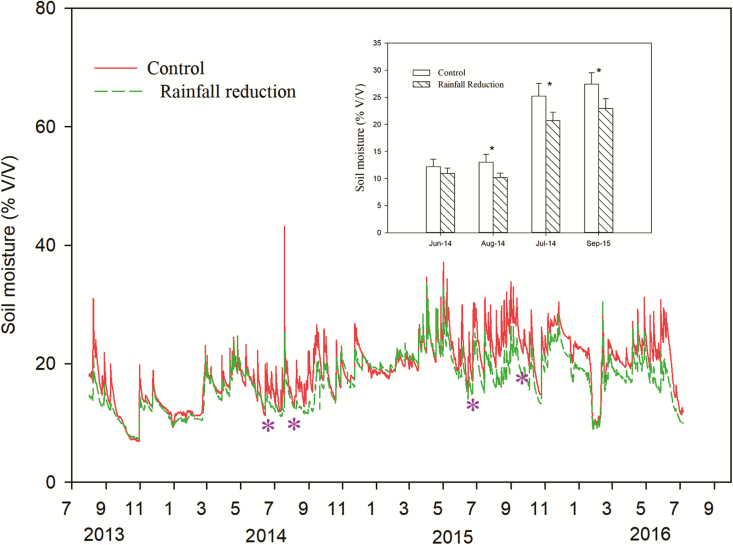
Soil moisture dynamics of control and rainfall-intercepted plots from August 2013 to July 2016. * refers to the date for measuring gas exchange, water potential and NSCs.

Marked fluctuations of soil moisture (by the volume percentage) in both plots occurred. Most of the time, soil moisture in the control plots was significantly higher than that in rainfall-intercepted plots (Fig. 1). There were almost no precipitations from May to the early June 2014, and in late June the soil moisture at top 10 cm layers was around 12 %. By the end of September 2016, all 35 *L. obtusiloba* trees in the rainfall-intercepted plots exhibited different degrees of dieback, while only five *L. obtusiloba* trees (of 47 total) in the control plots exhibited dieback. All *D. japonica* and *S. alnifolia* trees in all plots showed no signs of dieback (Table 1).

**Table 1.** Dieback rates of three species in control and rainfall-intercepted plots.

Species	Control plots			Rainfall-intercepted plots		
	Total	Dieback	Dieback rate	Total	Dieback	Dieback rate
*D. japonica*	27	0	0	28	0	0
*L. obtusiloba*	47	5	10.64 %	35	35	100 %
*S. alnifolia.*	29	0	0	30	0	0

### Stomatal response to dehydration

**Figure 2.**
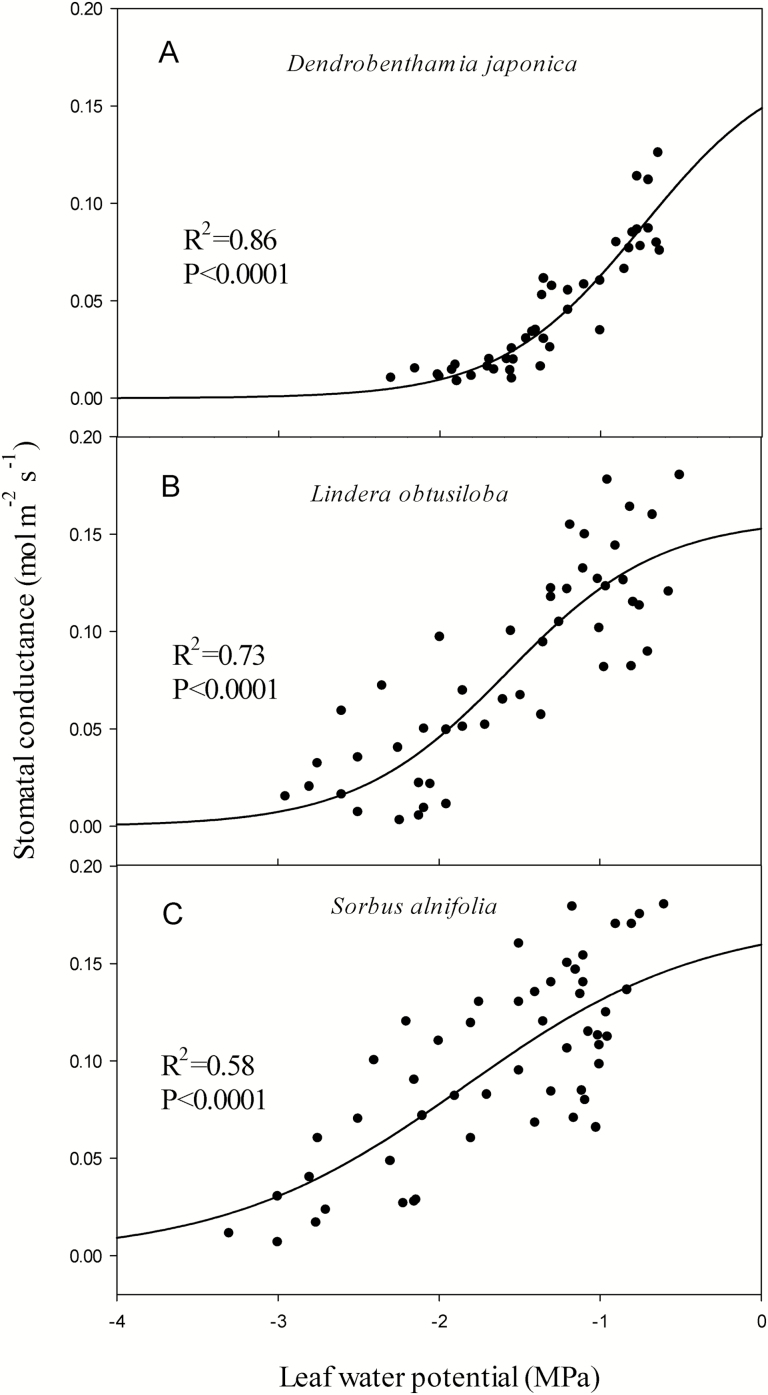
Leaf water potential plotted against stomatal conductance (*g*_s_) of the three species.

The three species differed in their degree of stomatal regulation of water potential, showing contrasting stomatal control strategies (Fig. 2). Stomatal conductance (*g*_s_) of *D. japonica* tended to reduce more abruptly when leaf water potential decreased than that of the other two species, with *S. alnifolia* displaying the slowest decreasing tendency. The water potentials at 50 % stomatal closure of *D. japonica*, *L. obtusiloba* and *S. alnifolia* were −0.88, −1.61 and −1.97 MPa, respectively, and the water potentials at complete stomatal closure (P_g88_) of *D. japonica*, *L. obtusiloba* and *S. alnifolia* were −1.70, −2.54 and −3.41 MPa, respectively. Regarding the degree of stomatal regulation of water potential, *D. japonica* was isohydric of the isohydric-anisohydric continuum, while *S. alnifolia* and *L. obtusiloba* were inclining to anisohydric. In view of the scatter observations for panels B and C of Fig. 2, a 90 percentile curve **[see****Supporting Information—Fig. S6****]** was made. The result showed that it had the same pattern over the three species and still provided the same interpretation as the original data did.

### Vulnerability curves

**Figure 3.**
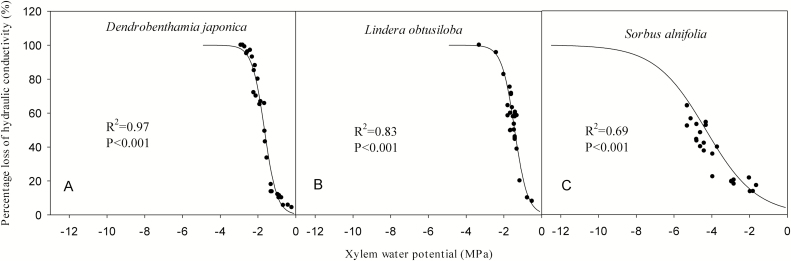
Curves of xylem vulnerability to cavitation of the three species.

The xylem VCs showed that *S. alnifolia* was less susceptible to cavitation than *D. japonica* and *L. obtusiloba* (Fig. 3). The P_88_ (water potential at 88 % loss in hydraulic conductivity) of *S. alnifolia* was −7.01 MPa, whereas that of *D. japonica* and *L. obtusiloba* was −2.31 and −2.11 MPa, respectively. The P_88_ of *S. alnifolia* was more than twice as negative as that of *D. japonica* and *L. obtusiloba* (Fig. 3; **see****Supporting Information—Fig. S5**). The P_50_ of *S. alnifolia*, *D. japonica* and *L. obtusiloba* was −4.3, −1.66 and −1.43 MPa, respectively.

### Stomatal safety margin and hydraulic safety margin

**Figure 4.**
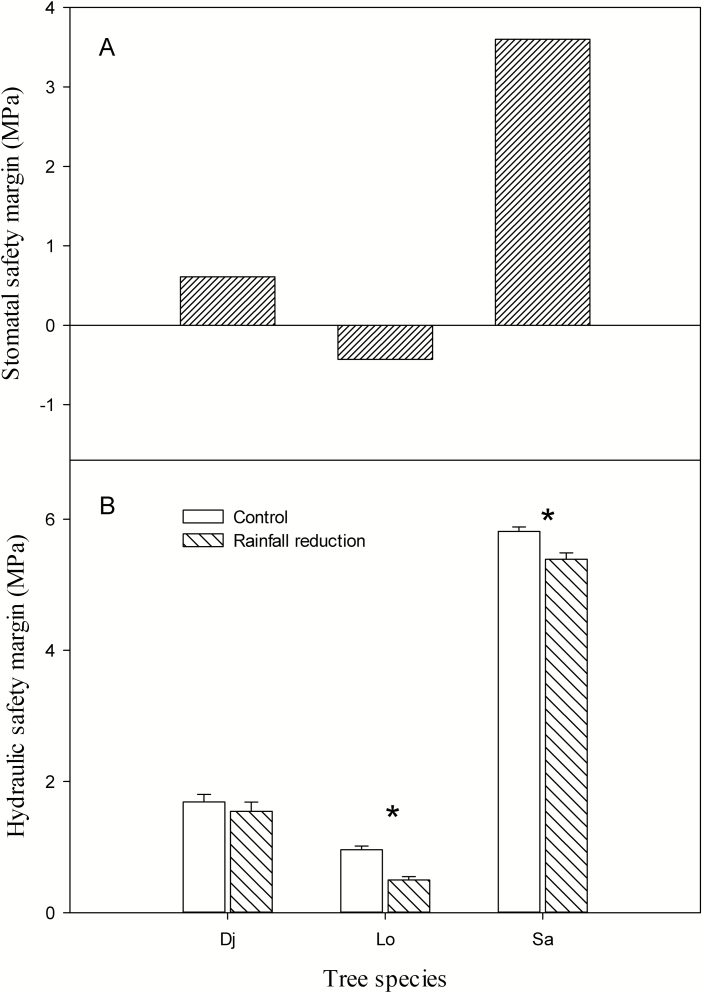
(A) Stomatal safety margin of the three species and (B) hydraulic safety margin of the three species in control and rainfall-intercepted plots. The difference between the minimum water potential (Ψ _min_) and P_88_ is regarded as hydraulic safety margin; the difference between P_g88_ and P_88_ is regarded as the stomatal safety margin. Star above bars indicates that hydraulic safety margin was significantly different between the control plots and the rainfall-intercepted plots, respectively (*P* < 0.05). Dj: *D. japonica*, Lo: *L. obtusiloba*, Sa: *S. alnifolia*.

Although the P_g88_ (water potential at stomatal closure) of *S. alnifolia* was the most negative among the three species, its stomatal closure occurred before the xylem lost 88 % hydraulic conductance; therefore, its stomatal safety margin (margin between P_g88_ and P_88_) was positive, while the stomatal safety margin of *L. obtusiloba* was negative. The stomatal safety margins of *D. japonica*, *L. obtusiloba* and *S. alnifolia* were 0.61, −0.43 and 3.60 MPa, respectively (Fig. 4A). As for P_50_, the stomatal safety margin was −0.46, −1.56 and 0.19 MPa, respectively, for *D. japonica*, *L. obtusiloba* and *S. alnifolia***[see****Supporting Information—Fig. S4A****]**. The hydraulic safety margin of *S. alnifolia* was the largest among the three species because its P_88_ was very negative, although its minimum water potential was also very negative. The hydraulic safety margin of *L. obtusiloba* was the lowest among the three species because its minimum water potential was also very negative, and the P_88_ of *L. obtusiloba* was the highest among the three species. The hydraulic safety margin of *D. japonica* was in between that of *L. obtusiloba* and *S. alnifolia* (Fig. 4B). When the hydraulic safety margin was calculated with P_50_, the pattern of the hydraulic safety margin over the three species was similar. The hydraulic safety margin of *L. obtusiloba* and *S. alnifolia* in rainfall-intercepted plots was also significantly lower than that their controls **[see****Supporting Information—Fig. S4B****]**. The turgor loss point (Ψ _TLP_) values of *D. japonica*, *L. obtusiloba* and *S. alnifolia* were −1.73 ± 0.033, −2.2 ± 0.058 and −2.67 ± 0.067 MPa, respectively, and P_g88_ showed a better linear relationship with Ψ _TLP_ compared with P_88_**[see****Supporting Information—Fig. S5****]**.

### Water potential

**Figure 5.**
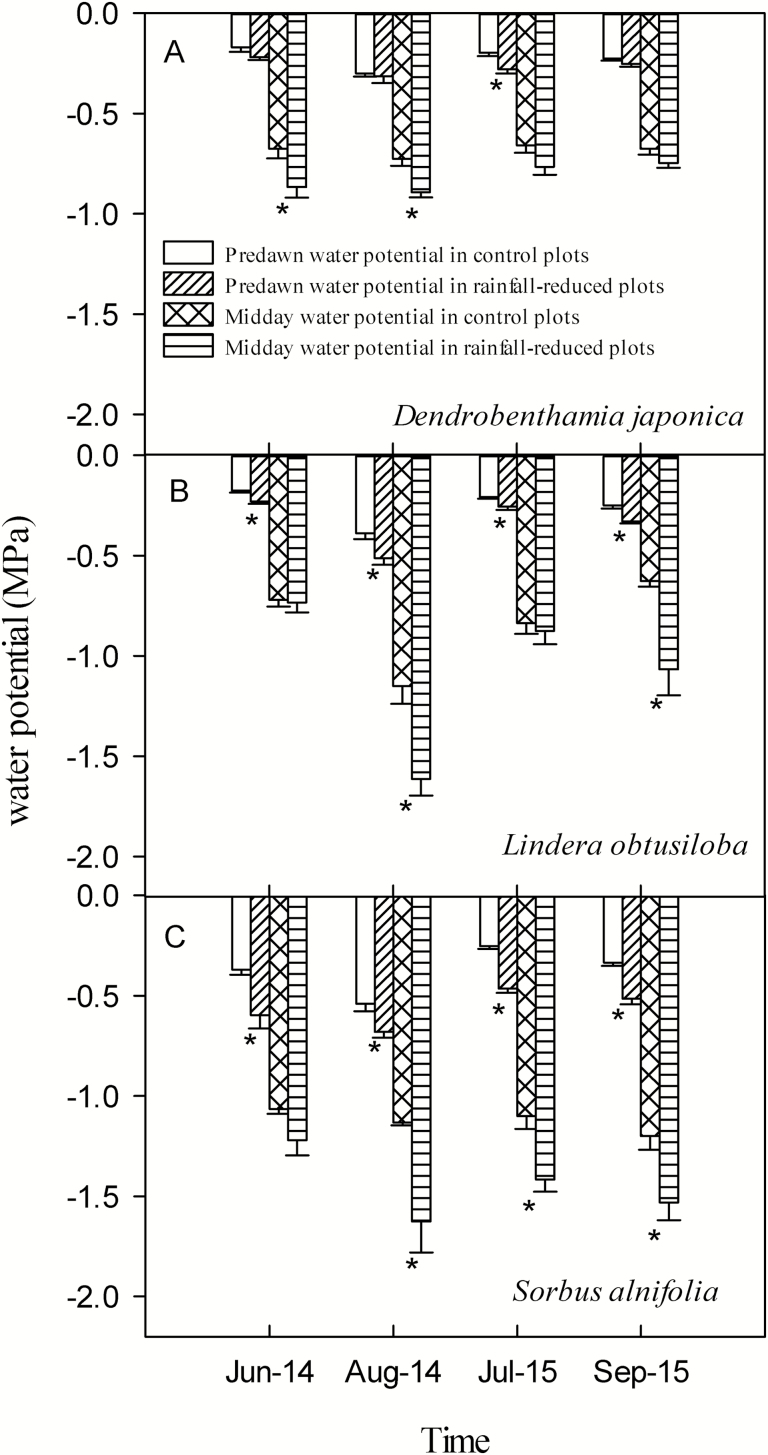
Predawn water potential and midday water potential of the three species in control and rainfall-intercepted plots. Means ± SE (*n* = 6) are shown. Star below bars indicates that predawn water potential and midday water potential were significantly different between the control plots and the rainfall-intercepted plots, respectively (*P* < 0.05).

*Dendrobenthamia japonica* in the rainfall-intercepted plots had once significantly lower predawn water potential than that in the control plots, and had twice significantly lower midday water potential out of four measurement occasions (Fig. 5; Table 2). In the same measurement occasions, *L. obtusiloba* in the rainfall-intercepted plots had twice significantly lower predawn water potential than that in the control plots, and had twice significantly lower midday water potential out of four measurement occasions. *Sorbus alnifolia* in the rainfall-intercepted plots had all significantly lower predawn water potential than that in the control plots, and had all significantly lower midday water potential of four measurement occasions. The differences between the control and rainfall-intercepted plots for *D. japonica* were lower than the other two species. Fluctuations in water potential of *D. japonica* at the four measurement times were also slightly less than those of the other two species. In contrast, the differences in both the predawn and midday water potentials of *S. alnifolia* between the control and rainfall-intercepted plots were higher than those of the other two species. Furthermore, fluctuations in water potential of *S. alnifolia* were also the largest of the three species. The water potential of *L. obtusiloba* was between that of *D. japonica* and *S. alnifolia*. Both the minimum predawn and midday water potentials of *S. alnifolia* were the lowest among the three species, and those of *D. japonica* were the highest. After rainfall reduction, the seasonal midday minimum water potentials (Ψ _min_) of *D. japonica*, *L. obtusiloba* and *S. alnifolia* were −0.892 ± 0.026, −1.613 ± 0.083 and −1.625 ± 0.155 MPa, respectively. All the minimum values were observed in August 2014 with leafy twigs, and used as the minimum water potentials for these species.

**Table 2.** Two-way ANOVA analysis results of variables of three understory species exposed to rainfall regimes. HSM refers to hydraulic safety margin; T * S refers to Treatment * Species; PWP refers to predawn water potential, and MWP to midday water potential; LNSC refers to leaf non-structural carbon, and XNSC to xylem NSC; DBH refers to diameter at breast height. Bold values indicate significant differences between treatments, among species, and treatments x species.

	Treatment		Species		T * S	
Variable	*F*	Sig.	*F*	Sig.	*F*	Sig.
HSM	51.73	**<0.01**	1201.67	**<0.01**	3.67	**0.038**
PWP2014/6	15.08	**<0.01**	44.67	**<0.01**	4.18	**0.027**
PWP2014/8	13.82	**<0.01**	47.77	**<0.01**	2.50	0.105
PWP2015/7	61.09	**<0.01**	28.28	**<0.01**	9.92	**<0.01**
PWP2015/9	53.46	**<0.01**	70.49	**<0.01**	11.64	**<0.01**
MWP2014/6	8.14	**<0.01**	37.44	**<0.01**	1.64	0.213
MWP2014/8	64.27	**<0.01**	69.84	**<0.01**	5.20	**0.012**
MWP2015/7	12.26	**<0.01**	59.16	**<0.01**	3.64	**0.039**
MWP2015/9	23.05	**<0.01**	46.41	**<0.01**	3.48	**0.044**
g_s_2014/6	11.72	**<0.01**	0.67	0.516	0.67	0.516
g_s_2014/8	24.48	**<0.001**	1.02	0.369	1.02	0.369
g_s_2015/7	0.02	0.883	0.34	0.710	0.34	0.710
g_s_2015/9	5.61	**0.022**	0.87	0.425	0.87	0.425
P_n_2014/6	4.18	**0.048**	0.84	0.438	0.84	0.438
P_n_2014/8	16.86	**<0.001**	0.43	0.653	0.43	0.653
P_n_2015/7	0.55	0.462	1.11	0.336	1.11	0.336
P_n_2015/9	19.44	**<0.001**	3.22	**0.049**	3.22	**0.049**
LNSC2014/6	1.11	0.302	2.24	0.129	12.74	**<0.001**
LNSC2014/8	0.96	0.336	50.15	**<0.001**	6.96	**<0.001**
LNSC2015/7	1.31	0.263	152.49	**<0.001**	3.29	0.053
LNSC2015/9	8.51	**<0.001**	219.71	**<0.001**	0.51	0.607
XNSC2014/6	12.25	**<0.001**	6.45	**<0.001**	21.41	**<0.001**
XNSC2014/8	3.90	0.057	85.58	**<0.001**	1.18	0.320
XNSC2015/7	34.38	**<0.001**	74.36	**<0.001**	0.65	0.531
XNSC2015/9	3.29	0.079	214.96	**<0.001**	18.87	**<0.001**
Length	19.85	**<0.001**	0.77	0.467	3.93	0.024
DBH	13.15	**<0.001**	13.47	**<0.001**	0.11	0.892
C13	0.03	0.863	107.70	**<0.001**	8.15	**<0.001**

### Gas exchange

**Figure 6.**
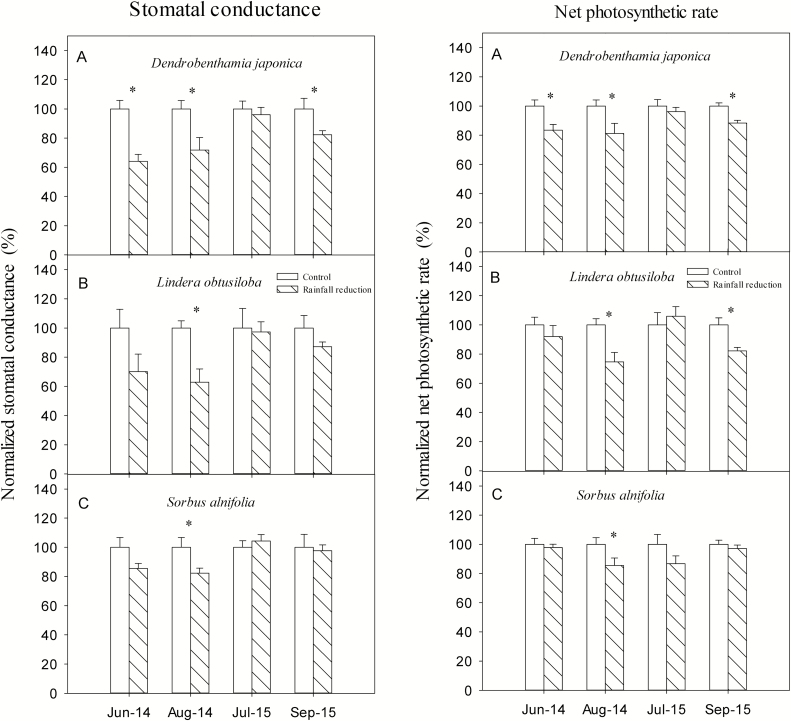
Stomatal conductance (*g*_s_) and net photosynthetic rate (*A*_n_) of the three species in control and rainfall-intercepted plots. The average *g*_s_ and *A*_n_ of the three species in the control plots were considered 100 %, and the numbers for the rainfall-intercepted plots showed the percentage relative to the control plots. Means ± SE (*n* = 6) are shown. Star above bars indicates a significant difference between the control plots and rainfall-intercepted plots (*P* < 0.05).

The *g*_s_ or *A*_n_ (net photosynthetic rate) of *D. japonica* in the rainfall-intercepted plots were three times significantly lower than that in the control plots out of four measurement occasions (Fig. 6; Table 2). *Lindera obtusiloba* in the rainfall-intercepted plots had twice significantly lower *g*_s_, and twice significantly lower *A*_n_ than that in the control plots out of four measurement occasions. *Sorbus alnifolia* in the rainfall-intercepted plots had no significantly differences in *g*_s_ and *A*_n_ from that in the control plots in all measurement occasions. In general, the difference between the control and rainfall-intercepted plots in terms of either *g*_s_ or *A*_n_ of *D. japonica* was highest among the three species. In order to observe how these responses are changing relative to water potentials, a graph with the absolute values was drawn. The result showed that there were obvious differences in *g*_s_ and *A*_n_ over the four observation occasions besides the treatments **[see****Supporting Information—Fig. S7****]**.

### Isotope composition and NSC concentration

**Figure 7.**
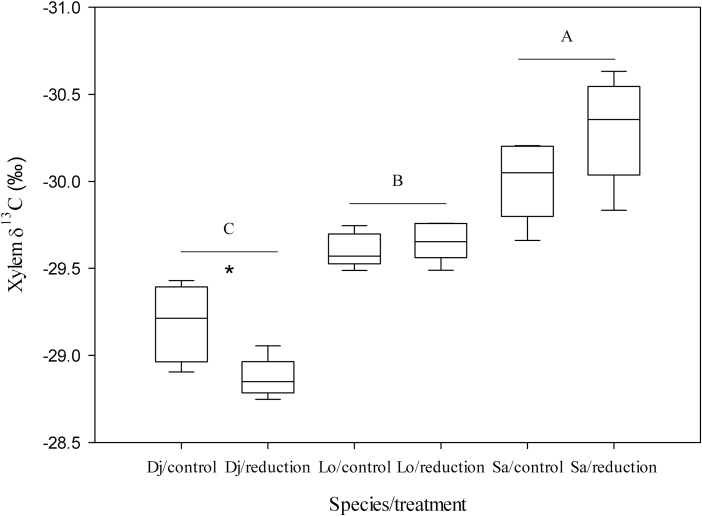
Xylem δ ^13^C of the three species in the control and rainfall-intercepted plots. Means ± SE (*n* = 6) are shown. Star above bars indicates that δ ^13^C was significantly different between the control and rainfall-intercepted plots (*P* < 0.05). Different letters indicate significant differences in δ ^13^C among the three species (*P* < 0.05). Dj: *D. japonica*, Lo: *L. obtusiloba*, Sa: *S. alnifolia*.

Regarding the δ ^13^C of xylem, only *D. japonica* displayed significant differences between the control and treatment plots. However, significant differences were observed in the xylem δ ^13^C among species regardless of the water regimes (Fig. 7; Table 2).

**Figure 8.**
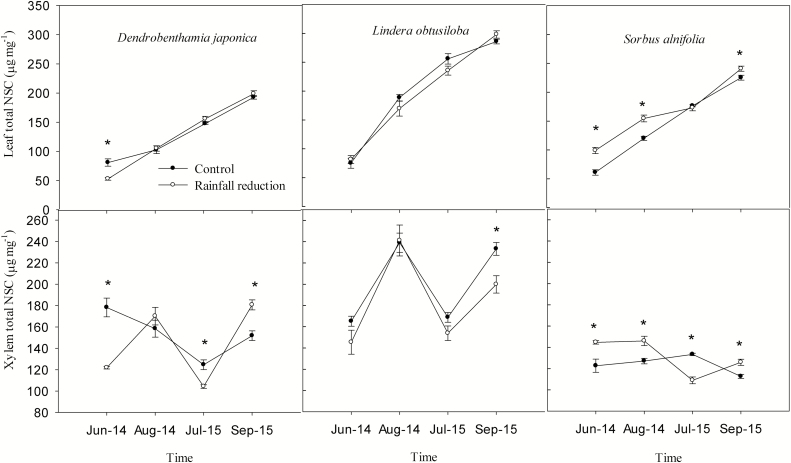
Total NSCs of the leaf and xylem of the three species in control and rainfall-intercepted plots. Means ± SE (*n* = 6) are shown. Asterisks above symbols indicates that NSC was significantly different between the control and rainfall-intercepted plots on the measurement occasion (P < 0.05).

The reduced-rainfall treatment significantly reduced the leaf NSC concentration of *D. japonica* in June 2014, and the xylem NSC concentration in June 2014 and July 2015 (Fig. 8; Table 2 and 3). In short, the reduced-rainfall treatment could have caused a drawdown of NSC stores of *D. japonica*, although its xylem NSC concentrations in rainfall-intercepted plots were significantly higher than those in control plots in September 2015. However, the leaf and xylem NSC concentration of *S. alnifolia* were significantly increased by the treatment in June 2014, August 2014 and September 2015, indicating that the reduced-rainfall treatment appeared to lead to an NSC increase in *S. alnifolia*, although its xylem NSC concentrations in the control plots were significantly higher than those in the reduced-rainfall plots in July 2015.

**Table 3.** Differences in percentage of NSC between rainfall reduction and control.

	Leaf			Xylem		
	*D. japonica*	*L. obtusiloba*	*S. alnifolia*	*D. japonica*	*L. obtusiloba*	*S. alnifolia*
June 2014	65.4399	108.5152	162.4572	68.38887	88.05862	117.9078
August 2014	102.2679	89.94911	128.7061	107.3747	100.8574	114.8837
July 2015	105.6234	92.0693	98.10348	83.65415	91.15034	81.72277
September 2015	103.138	104.277	106.8595	118.9517	85.65916	111.7856

### Growth

**Figure 9.**
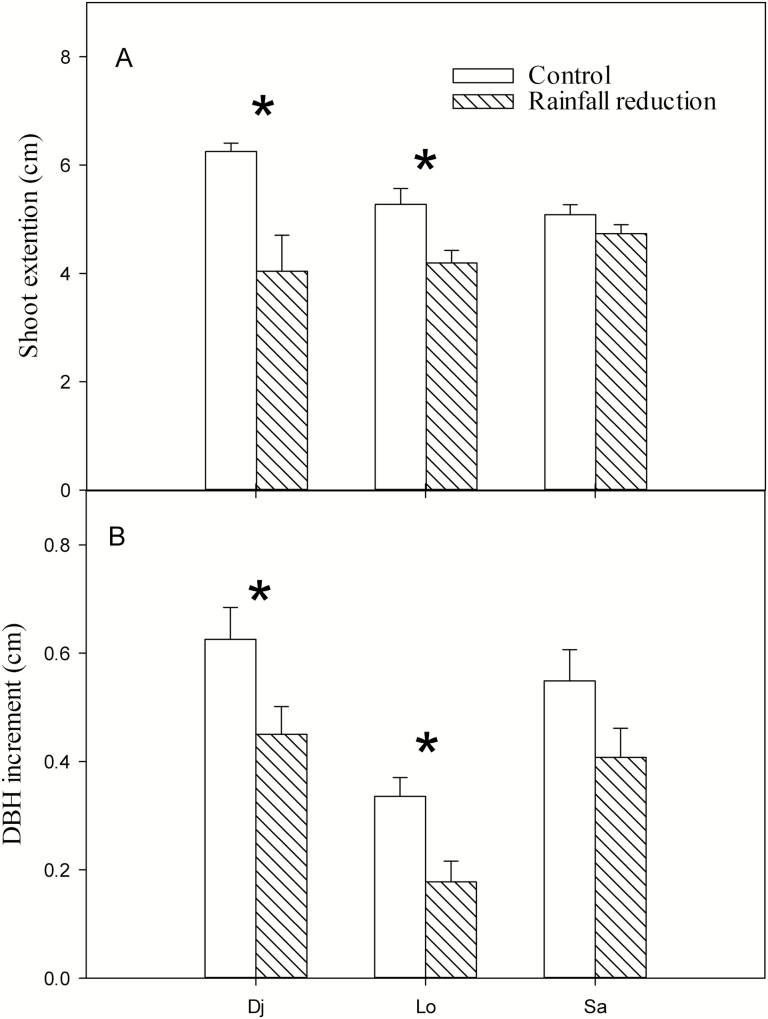
(A) Shoot extensions (n = 6) and (B) diameter at breast height (DBH) increments (n = 24–40) of three species in control and rainfall-intercepted plots. Means ± SE are shown. *above columns indicates that DBH increments or shoot extensions were significantly different between the control and rainfall-intercepted plots (*P* < 0.05). Dj: *D. japonica*, Lo: *L. obtusiloba*, Sa: *S. alnifolia*.

The reduced-rainfall treatment significantly reduced shoot extensions of *D. japonica* and *L. obtusiloba* as well as DBH increments, but no significant differences were detected between the control and treatment regarding shoot extensions and DBH increments of *S. alnifolia* (Fig. 9; Table 2).

## Discussion

### Stomatal sensitivity associated with NSC concentration and growth

The observed trends in water potential, *g*_s_, xylem vulnerability and NSCs in these three co-occurring sub-canopy tree species suggest that their strategies for stomatal behaviour and their xylem hydraulic systems varied. In rainfall reduction treatment, *L. obtusiloba* suffered from dieback, while *D. japonica* and *S. alnifolia* did not. Although *D. japonica* and *S. alnifolia* did not show dieback, their NSC and growth had differential responses dependent on the stomatal regulation. This different stomatal regulation of water potential among species resulted in photosynthetic carbon fixation being more sensitive to drought in *D. japonica* than in *S. alnifolia*. After rainfall reduction, the limitation of gas exchange was greater in isohydric *D. japonica* than anisohydric *S. alnifolia*; therefore, the net photosynthetic rate of *S. alnifolia* was less affected by rain-exclusion treatment. Moderate water stress is generally associated with an increase in NSCs, resulting from decreased plant growth before photosynthesis during water stress (McDowell 2011; Woodruff and Meinzer 2011; Woodruff *et al.* 2015). In addition, limitations in phloem transport under drought stress can result in a reduction in the NSC export capacity from source to sink, which can also lead to an increase of NSCs in the aerial parts of plants. For anisohydric *S. alnifolia*, NSC accumulation in the rainfall-intercepted leaf and xylem was more than that in the control for three occasions out of four measurements in two growth seasons, but the rainfall-intercepted treatment reduced the leaf and xylem NSC concentrations in the more isohydric *D. japonica*. As a whole, the water potential was more negative in anisohydric *S. alnifolia* than in isohydric *D. japonica*, but the constraints on carbon accumulation were lower in *S. alnifolia*. When water stress is relieved, the previous storage of NSCs is used for plant growth (Woodruff *et al.* 2015). In July 2015, which was the peak period of plant growth in the Baotianman Mountains region and was wetter than June 2014 and August 2014, the leaf and xylem of *S. alnifolia* did not have an NSC accumulation in the reduced-rainfall plots compared with that of the leaf and xylem in the control plots, which suggested more NSCs might be used to grow. In addition, the Baotianman Mountains were frequently wet during growing season. Most of rainfalls occurred in the growing season, accounting for 62–77 % of annual precipitation in 2014 and 2015. As expected, in the rainfall reduction plots, *S. alnifolia* maintained its growth in the long term, while the growth of *D. japonica* decreased, which agrees with the theory: anisohydric species tend to accumulate NSCs for maintaining their long-term growth, and isohydric species are sensitive to drought conditions by reducing NSC accumulation, resulting in reduced growth over the long term. Thus, the isohydric/anisohydric degree was associated to NSC status and growth of plants. However, the anisohydric-inclining *L. obtusiloba* also reduced its growth, despite without change in NSC, probably due to frequent diebacks.

### Synergistic effect of stomatal sensitivity and xylem vulnerability on drought resistance of plants

*Dendrobenthamia japonica* is isohydry in iso-anisohydric continuum, while the other two species are inclining to anisohydry. *Dendrobenthamia japonica* and *L. obtusiloba* are vulnerable to xylem cavitation, while *S. alnifolia* is relatively resistant to xylem cavitation. Unlike juniper in Piñon–juniper woodlands of southwestern USA (Dickman *et al.* 2015), the near-anisohydric *L. obtusiloba* was not associated with high resistant xylem cavitation; to the contrary, it was vulnerable to xylem cavitation. As a result, the species was not able to tolerate the precipitation reduction-caused drought, even mild drought, which is consistent to previous observations showing that anisohydric ericoid shrubs in South Africa suffered considerable reductions in growth and flowering and increased mortality in drought condition (West *et al.* 2012). Although *D. japonica* was also vulnerable to xylem cavitation, it, with its isohydric trait, could timely close its stomata in response to drought stress to prevent from building up water tension in the vessels. Thus, the species was not impacted by the mild drought stress very much in this study. The third species of *S. alnifolia* used another strategy to handle the drought stress, and it is anisohydric, even more than *L. obtusiloba*, but it has relatively stronger resistance to xylem cavitation, like *Juniperus monosperma* (Dickman *et al.* 2015). Therefore, it also successfully survived to the mild drought stress.

From the responses of the three species, it can be concluded that either isohydric vulnerability to cavitation or anisohydric resistance to cavitation all has capacity to tolerate drought stress, at least mild drought stress in this experiment. In contrast, the anisohydric vulnerability cannot survive drought environment, even mild drought condition. It is thus clear that *L. obtusiloba* can be sensitive to climate change-caused drought although still in sub-humid areas. Recently, Hochberg *et al.* (2018) have pointed out that the iso/anisohydric alone does not determine which type of species is suitable for wetness, and which type is suitable for drought. Our research also proves that the iso/anisohydric is not directly related to drought resistance; instead, the interaction of stomatal behaviour and vulnerability to xylem cavitation plays a crucial role. Moreover, the rooting depth also plays a role for plant to deal with drought stress. In this study, *S. alnifolia* is the only species that had different predawn pressures between treatments for all four measurement days, suggesting it probably has the shallower root system. And the pressure drop between predawn and midday was maintained for rain exclusion. On the other hand, *D. japonica* only showed a significant difference predawn between treatments out of the four measurement days, and it actually did not show any difference for midday water potentials between treatments. This species was more hydrated than the other species, indicating that this species had a deeper rooting system that was obtaining moisture for soil levels, and hence was not affected by the rain-exclusion treatment. *Lindera obtusiloba* was intermediate. In addition both *S. alnifolia* and *L. obtusiloba* were more dehydrated in midday, reflecting that their stomata maintained more aperture than *D. japonica*.

Previous studies have suggested that isohydric plants can experience higher mortality and die from carbon starvation and that anisohydric plants are likely to die from hydraulic failure during extreme droughts (McDowell *et al.* 2008; Dickman *et al.* 2015; Woodruff *et al.* 2015). We found that near-anisohydric *L. obtusiloba*, whose P_g88_ was between that of *D. japonica* and *S. alnifolia*, displayed severe dieback after the rainfall reduction treatment, even under a mild drought stress. Placement of Ψ _min_ in the sequence of threshold values of water potential (Ψ _min_, P_g88_, P_88_) indicates the drought responses of plants under seasonal water stress in natural conditions (Bartlett *et al.* 2016). The margin between Ψ _min_ and P_g88_ or P_88_ may determine whether the plant suffers carbon starvation or hydraulic failure under water stress, in which a large positive margin implies a relatively conservative response and a small margin (or even a negative margin) suggests a risky response (Skelton *et al.* 2015). The xylem vulnerability of *L. obtusiloba* was the greatest among the three species, and its P_g88_ was quite negative, and its Ψ _min_ was also relatively negative because of its stomatal behaviour (much more negative than that of *D. japonica*); therefore, the hydraulic safety margin of *L. obtusiloba* was the lowest among the three species. In terms of the stomatal safety margin (margin between P_g88_ and P_88_), only *L. obtusiloba* had negative stomatal safety margin among the three species. The P_g88_ (water potential at stomatal closure) of *L. obtusiloba* was lower than the P_88_, that is, its stomatal closure occurred after the xylem lost 88 % hydraulic conductance; thus, its stomatal regulation could not efficiently protect the xylem vessels from hydraulic dysfunction. In addition, the Ψ _min_ of *L. obtusiloba* was not lower than its P_g88_, and the leaf and xylem NSC concentrations of *L. obtusiloba* in the reduced-rainfall plots were similar to those in the control plots. Therefore, the dieback of *L. obtusiloba* should result from hydraulic failure. While the Ψ _min_ of both *D. japonica* and *S. alnifolia* was higher than their P_88_, their hydraulic safety margin was positive and relatively higher, suggesting that the two species did not suffer hydraulic failure. In comparison between *D. japonica* and *S. alnifolia*, there were obvious differences in both hydraulic safety margin and stomatal safety margin, suggesting that *D. japonica* would be more susceptible to drought stress than *S. alnifolia* did.

Stomatal closure is considered to be associated with the turgor loss point (Ψ _TLP_) during drought (Brodribb and Holbrook 2003). Our results showed that P_g88_ displayed a linear relationship with Ψ _TLP_ across the three species **[see****Supporting Information—Fig. S5****]**. All Ψ _TLP_ values of the three species occurred before P_g88_, which is similar to the observations by Bartlett *et al.* (2016). These results suggested that turgor loss would induce stomatal closure and indicated that plants would not undergo stomatal closure at high water potentials to prevent wilting. Unlike the relationship between Ψ _TLP_ and P_g88_, the P_g88_ and P_88_ were not linearly related with each other, which is consistent to the results of previous studies (Skelton *et al.* 2015). Similar to the explanation provided by Skelton *et al.* (2015), stomatal closure might be entirely unrelated to xylem hydraulic conductance, which contrasts with the functional role of stomatal closure in avoiding xylem cavitation, or a non-linear relationship exists between P_g88_ and P_88_ that is influenced by multiple factors.

### Stomatal sensitivity and δ^13^C

Stable carbon isotope ratios (δ ^13^C) of tree wood or foliage can be used to evaluate the degree of openness of the stomatal aperture of different tree species or under different water conditions because δ ^13^C is correlated with leaf-level physiological processes. Discrimination against ^13^C occurs during photosynthesis, which is proportional to the partial pressure of CO_2_ inside the leaf (Farquhar *et al.* 1982). Therefore, an increase in δ ^13^C of tree wood or foliage indicates that *g*_s_ is lower for long term in different tree species or under different water conditions. In this study, the δ ^13^C of xylem of tree shoots of the three species was in the order of *D. japonica* > *L. obtusiloba* > *S. alnifolia*, and only the δ ^13^C value of *D. japonica* displayed significant difference between the control and reduced-rainfall treatment, supporting that *D. japonica* was more dependent on stomatal regulation. Therefore, based on the above results, regarding the degree of stomatal regulation of water potential, *D. japonica* and *S. alnifolia* occupied very different positions on the iso-anisohydric continuum of stomatal regulation, while *L. obtusiloba* was in the middle.

## Conclusion

In a rainfall reduction experiment, both the anisohydric *S. alnifolia* with strong resistance to cavitation and isohydric *D. japonica* though high vulnerability did not exhibit hydraulic failure, but near-anisohydric *L. obtusiloba* with vulnerable xylem was highly susceptible to drought dieback that resulted from hydraulic failure. This study revealed that hydraulic failure was also associated with the hydraulic safety margin (the difference between Ψ _min_, which was affected by the stomatal closure, and xylem hydraulic threshold (e.g. P_88_), representing vulnerability to water deficit). Among the three species, the hydraulic safety margin of *L. obtusiloba* was the smallest, especially in the rainfall-reduced plots. Of the two species without dieback, their sensitivity to drought also followed the hydraulic safety margin, and rainfall reduction decreased growth of *D. japonica*, but did not influence growth of *S. alnifolia*; meanwhile, rainfall reduction led to a decrease of NSCs in *D. japonica*, but an increase in *S. alnifolia*. Thus, we conclude that the stomatal safety margin and the hydraulic safety margin can interpret the sensitivity of the three sub-canopy species to drought. *Lindera obtusiloba* could be sensitive to climate change-caused drought although still in sub-humid areas, and its drought-induced tree dieback is determined by the interaction of stomatal behaviour and xylem vulnerability. The different stomatal regulation of water potential results in differential responses in NSC and growth of *D. japonica* and *S. alnifolia*.

## Supporting Information

The following additional information is available in the online version of this article—

Figure S1. Image of the precipitation-reduced plots (~50 % of the plot area was covered with interception roof).

Figure S2. Image of the dieback of *Lindera obtusiloba.*

Figure S3. The pressure-volume curves for each species are presented as a supplementary material.

Figure S4. (A) Stomatal safety margin of the three species and (B) hydraulic safety margin of the three species in control and rainfall-intercepted plots.

Figure S5. Water potential at stomatal closure (P_g88_) plotted against water potential at the turgor loss point (Ψ _TLP_) (A) and P_88_ (B) of the three species.

Figure S6. Leaf water potential plotted against stomatal conductance (*g*_s_) of the three species.

Figure S7. Stomatal conductance (*g*_s_) and net photosynthetic rate (*A*_n_) of the three species in control and rainfall-intercepted plots.

plz058_suppl_Supporting_Information
