# Supplementary material for: Interaction of stomatal behaviour and vulnerability to xylem cavitation determines the drought response of three temperate tree species
Source: AoB Plants. 2019 Sep 23;11(5):plz058. doi: 10.1093/aobpla/plz058 (PMC6802943; doi:10.1093/aobpla/plz058)
Supplement: plz058_suppl_Supporting_Information [file plz058_suppl_supporting_information.pdf]

Supplementary Figures

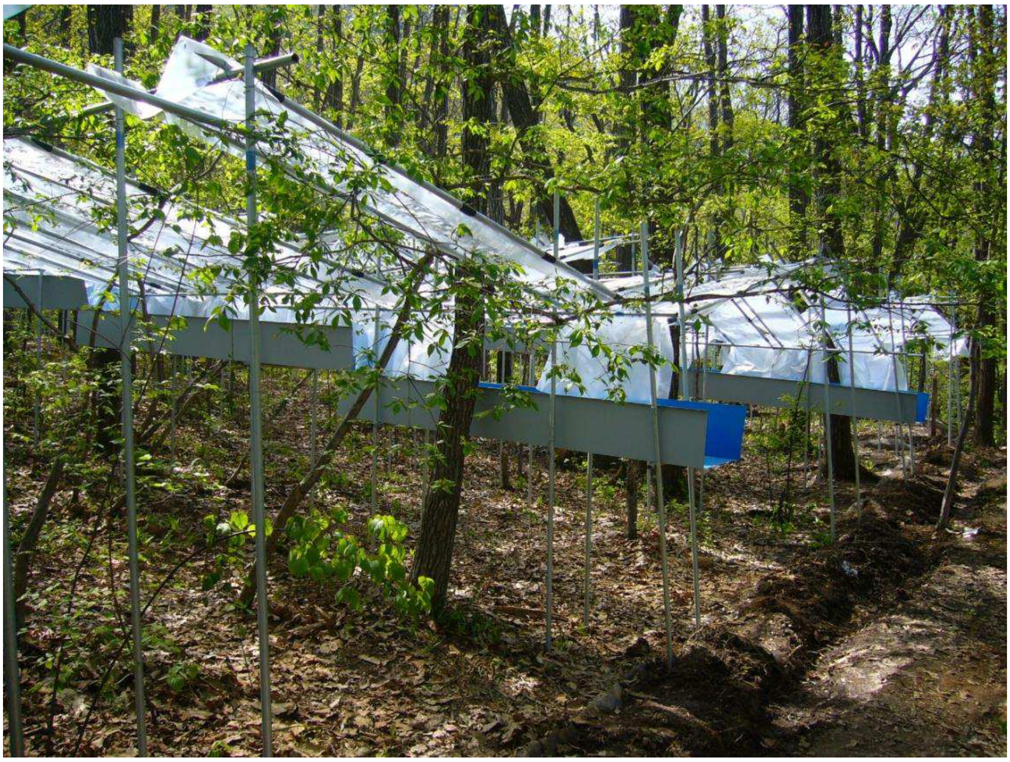

Supplemental Figure 1. Image of the precipitation reduced plots (about 50% of the plot area was covered with interception roof).

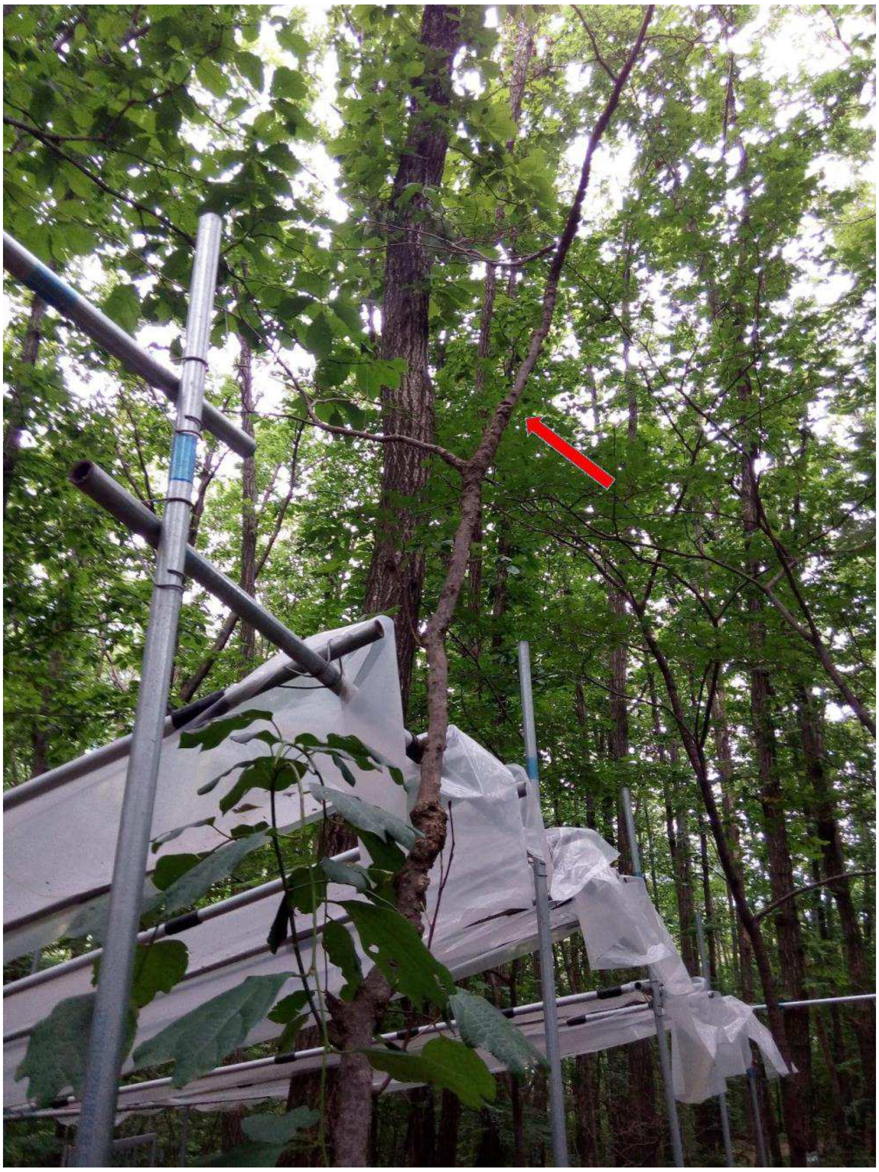

Supplemental Figure 2. Image of the dieback of *Lindera obtusiloba*.

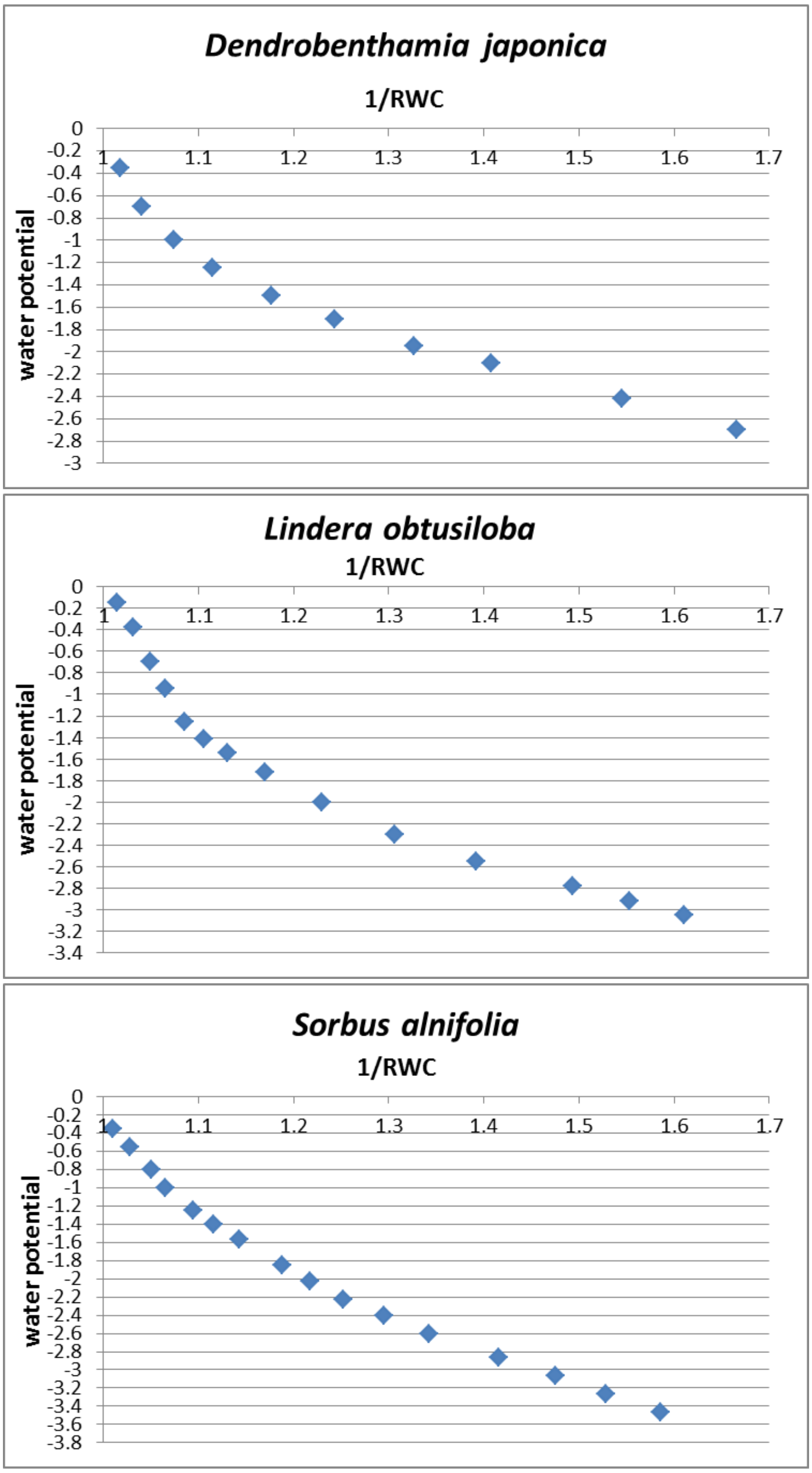

Supplemental Figure 3. The pressure volume curves for each species are presented as a supplemental material.

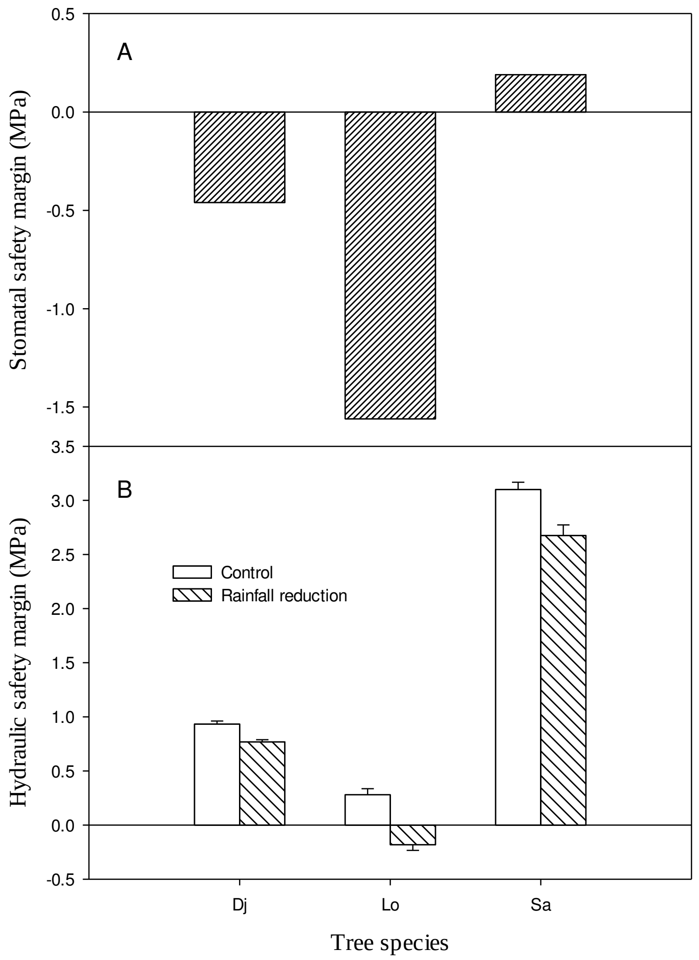

Supplemental Figure 4 (A) Stomatal safety margin of the three species and (B) hydraulic safety margin of the three species in control and rainfall-intercepted plots. Dj: *D. japonica*, Lo: *L. obtusiloba*, Sa: *S. alnifolia.* The difference between the typical minimum water potential (Ψ_{min}) and P_{50} is regarded as hydraulic safety margin; the difference between P_{g88} and P_{50} is regarded as the stomatal safety margin.

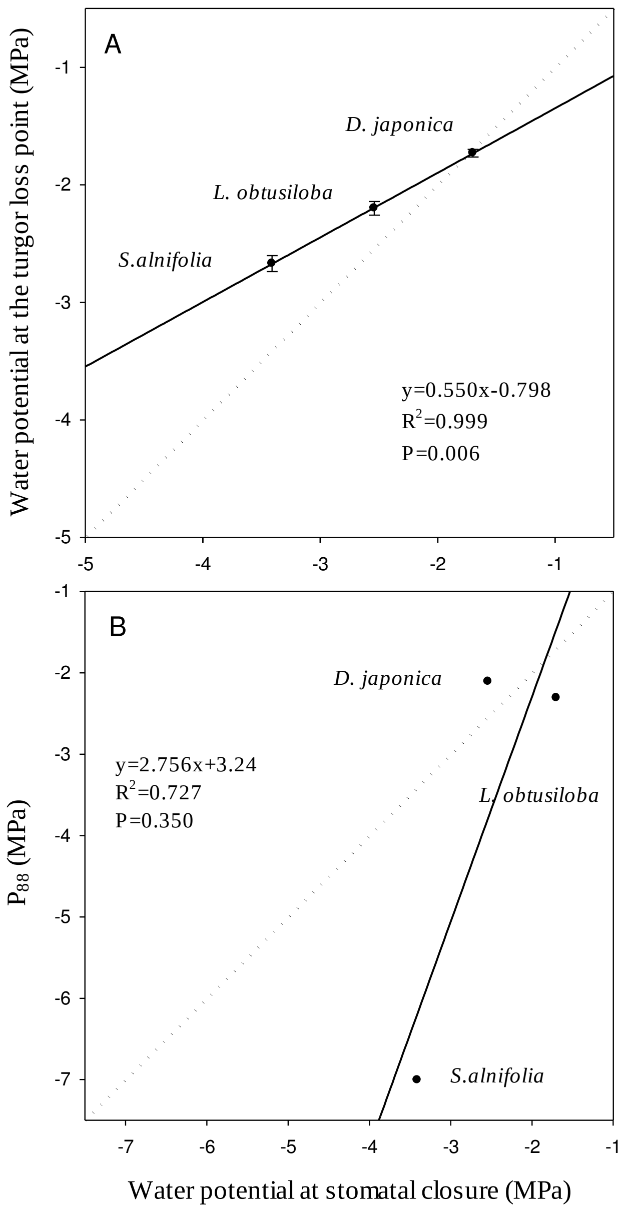

Supplemental Figure 5. Water potential at stomatal closure (P_{g88}) plotted against water potential at the turgor loss point (Ψ_{TLP}) (A) and P_{88} (B) of the three species. The solid lines represent regression lines and dash lines represent 1:1 line.

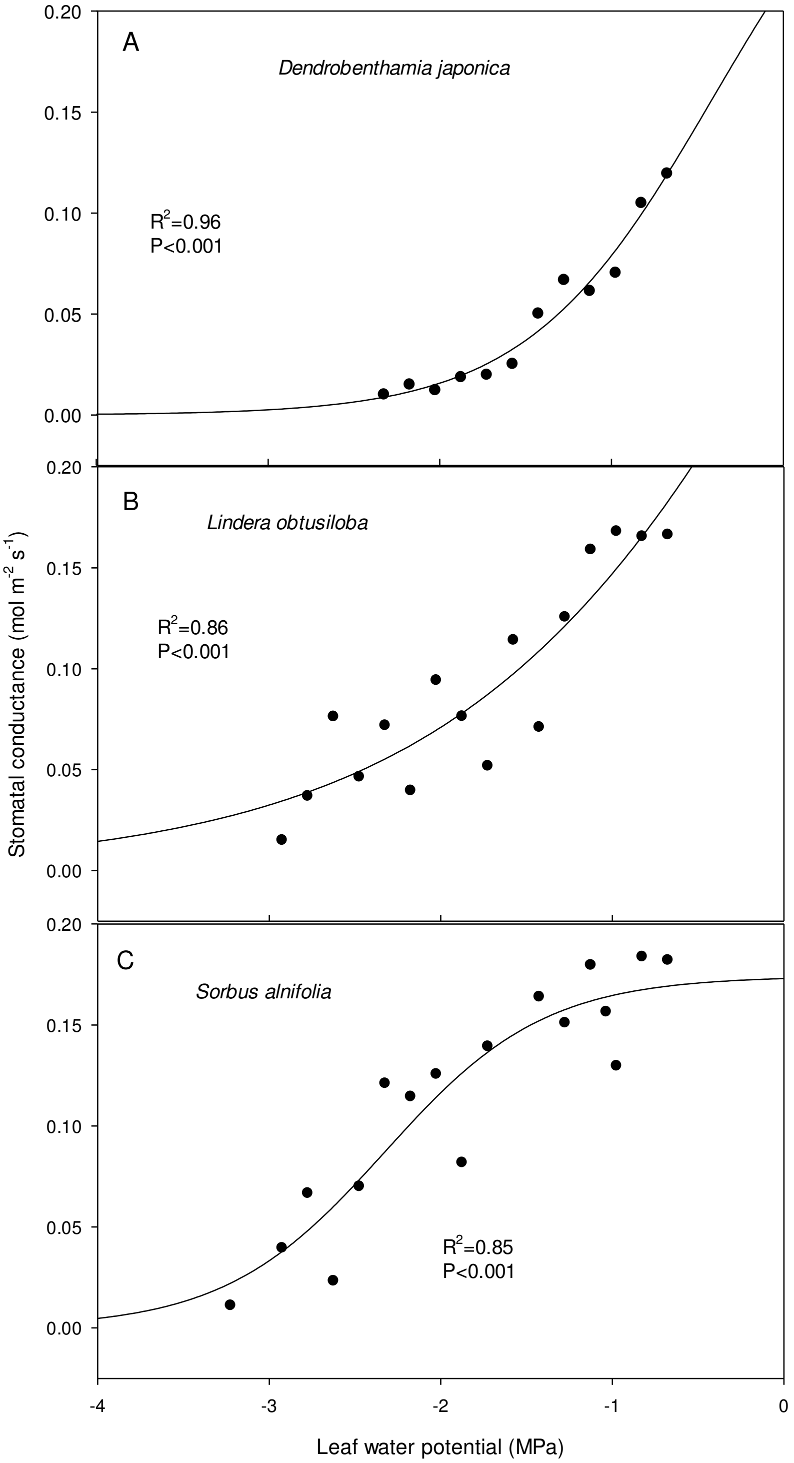

Supplemental Figure 6. Leaf water potential plotted against stomatal conductance (g_s) of the three species. The leaf water potential is used as the fixed factor, ranked, and separated by an interval of 0.15 MPa. The 90 percentile of g_s in each interval is calculated and fitted to a 90 percentile curve.

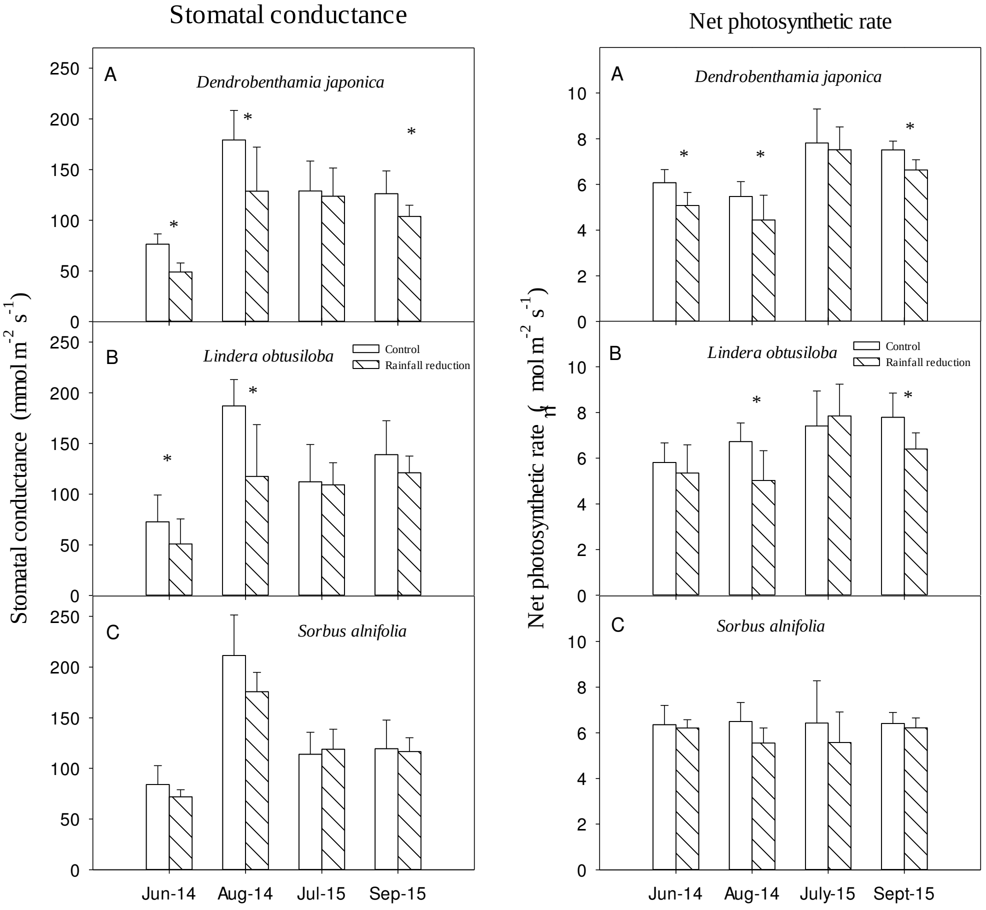

Supplemental Figure 7. Stomatal conductance (g_s) and net photosynthetic rate (A_n) of the three species in control and rainfall-intercepted plots. Means ± SE (n = 6) are shown. Star above bars indicates a significant difference between the control plots and rainfall-intercepted plots ($p < 0.05$).
